# Predicting early mortality and severe intraventricular hemorrhage in very-low birth weight preterm infants: a nationwide, multicenter study using machine learning

**DOI:** 10.1038/s41598-024-61749-1

**Published:** 2024-05-12

**Authors:** Yun-Hsiang Yang, Ts-Ting Wang, Yi-Han Su, Wei-Ying Chu, Wei-Ting Lin, Yen-Ju Chen, Yu-Shan Chang, Yung-Chieh Lin, Chyi-Her Lin, Yuh-Jyh Lin

**Affiliations:** 1 Department of Pediatrics, National Cheng Kung University Hospital, College of Medicine, National Cheng Kung University, No.138, Sheng Li Road, Tainan, Taiwan; 2 Department of Pediatrics, Ditmanson Medical Foundation Chia-Yi Christian Hospital, Chia-Yi, Taiwan; 3 Department of Emergency Medicine, Chi Mei Medical Center, Tainan, Taiwan; 4 Department of Pediatrics, E-Da Hospital, I-Shou University, Kaohsiung, Taiwan

**Keywords:** Medical research, Risk factors

## Abstract

Our aim was to develop a machine learning-based predictor for early mortality and severe intraventricular hemorrhage (IVH) in very-low birth weight (VLBW) preterm infants in Taiwan. We collected retrospective data from VLBW infants, dividing them into two cohorts: one for model development and internal validation (Cohort 1, 2016–2021), and another for external validation (Cohort 2, 2022). Primary outcomes included early mortality, severe IVH, and early poor outcomes (a combination of both). Data preprocessing involved 23 variables, with the top four predictors identified as gestational age, birth body weight, 5-min Apgar score, and endotracheal tube ventilation. Six machine learning algorithms were employed. Among 7471 infants analyzed, the selected predictors consistently performed well across all outcomes. Logistic regression and neural network models showed the highest predictive performance (AUC 0.81–0.90 in both internal and external validation) and were well-calibrated, confirmed by calibration plots and the lowest two mean Brier scores (0.0685 and 0.0691). We developed a robust machine learning-based outcome predictor using only four accessible variables, offering valuable prognostic information for parents and aiding healthcare providers in decision-making.

## Introduction

The first week of life is considered the most vulnerable period for newborns in terms of mortality, particularly among very low birth weight (VLBW) preterm infants^1^. Despite surviving these critical initial weeks following birth, VLBW preterm infants remain at a heightened risk for adverse long-term neurodevelopmental outcomes^2^. This risk can be primarily attributed to intraventricular hemorrhage (IVH), with approximately 95% of IVH cases occurring during this period^3^.

The significance of a dependable and timely risk assessment tool for early mortality and incidence of severe IVH cannot be overstated. This tool could not only provide a structured framework for parents and healthcare providers during the decision-making process but also offer valuable insights into recommending appropriate levels of care based on estimations of mortality and poor outcomes. For example, patients with a high probability of severe IVH may require tailored circulatory management strategies^4^. Moreover, the early identification of infants at the highest risk of developing severe IVH holds promise for enhancing the design of future clinical studies and optimizing the selection of participants for trials^5^.

In Taiwan, the incidence of preterm births has gradually increased from 8.85% in 2004 to 10.73% in 2014, a trend observed on a global scale^6^ Notably, the preterm birth rate in Taiwan has surpassed that in most OECD countries^7^ However, to the best of our knowledge, the existing literature has only identified certain risk factors associated with mortality and severe IVH in Taiwan^8–10^ The establishment of a nationwide outcome predictor applicable for the Taiwanese population remains an unmet need.

Therefore, this study aimed to develop and validate a straightforward machine learning (ML)-based outcome estimator, utilizing readily available data shortly after birth, to predict the probability of early mortality and development of severe IVH in VLBW preterm infants.

## Methods

### Study design and population cohorts

In this retrospective observational study, cohort data of VLBW preterm infants was obtained from the Taiwan Neonatal Network, established in 2016 to compile nationwide clinical data of preterm infants delivered in Taiwan from 33 medical centers. The enrollment criteria outlined by the Taiwan Neonatal Network include live-born infants born in Taiwan, with birth weights ranging from 401 to 1500 g or gestational ages ranging from 22 weeks 0 days to 29 weeks 6 days. This data was then used to establish and investigate two cohorts.

Cohort 1 comprised infants born between 2016 and 2021. Their data were collected for subsequent model development, internal validation and model comparison. Cohort 2 comprised infants born in 2022 and was included in the external validation.

The inclusion criteria were gestational age (GA) between 22 weeks and 0 days to 36 weeks and 6 days and a birth body weight (BBW) of less than 1500 g. Infants with missing data were excluded.

This study has been approved by the National Cheng Kung University Hospital Institutional Review Board (A-ER-111–115). The need of informed consent was waived by the National Cheng Kung University Hospital Institutional Review Board due to the fact that data were anonymized and de-identified. All methods were performed in accordance with the relevant guidelines and regulations.

### Outcomes

The primary outcomes of the study included: early mortality, severe IVH, and early poor outcomes (early mortality or severe IVH). Early mortality was defined as death within the first week of life and severe IVH was defined as IVH grade III or IV on cranial ultrasound, graded using Volpe’s grading system^11^

### Data preprocessing

We collected essential data as variables for each enrolled infant, resulting in a total of 23 variables. These variables included the following: antenatal steroid use; prenatal magnesium sulphate (MgSO4) use; pregnancy-induced hypertension; chorioamnionitis; GA; BBW; multiple births; Cesarean section; small for GA (defined as birth weight below the 10th percentile for GA, referencing values for birth weight distributions from a previous study of the Taiwanese population)^12^; sex; 1-min Apgar score; 5-min Apgar score; body temperature (defined as the rectal temperature measured for the first time within the first hour of birth); early-onset sepsis (defined as culture-proven sepsis occurring within 72 h of birth); respiratory distress syndrome; congenital anomalies (including chromosomal anomalies, skeletal dysplasia, inborn errors of metabolism, lethal or life-threatening anomalies in the cardiovascular, gastrointestinal, genitourinary, or pulmonary system, and other lethal or life-threatening anomalies); and seven delivery room resuscitation managements, including, neonatal resuscitation, oxygen supplementation, delivery room continuous positive airway pressure ventilation, positive pressure ventilation, endotracheal tube ventilation, chest compressions, and epinephrine administration. RapidMiner software version 10.0 (Altair Engineering, Troy, MI, USA; www.rapidminer.com) was used for data input and the cleaning of missing data.

### Selection of variables

To facilitate practical applicability, we conducted variable selection using the information gain attribute evaluator provided by Weka software version 3.8.6 (Waikato Environment for Knowledge Analysis, Hamilton, New Zealand). After measuring the entropy gain in relation to the outcomes, an information gain attribute evaluator was used to evaluate the significance of each of the 23 variables^13^ Additionally, we conducted an evaluation of collinearity between each variable. In the interest of establishing a more streamlined model, we selected the top-ranked variables based on their ranking.

### ML algorithms and model building

**Figure 1:**
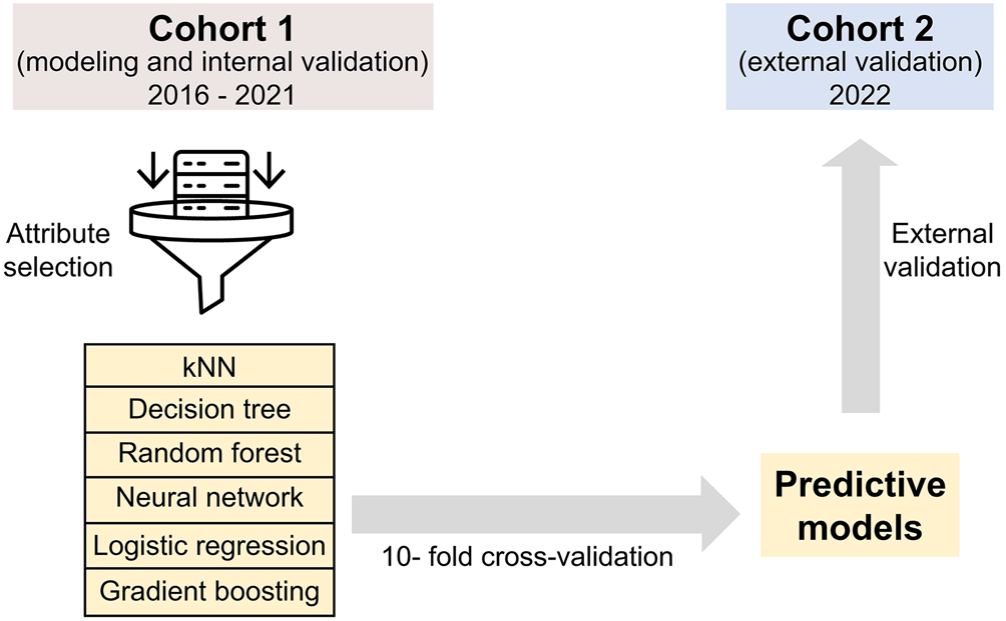
Flowchart of machine learning to build the predictive model.

The flow chart for building models using ML algorithms via Orange software version 3.34.0 (University of Ljubljana, Ljubljana, Slovenia)^14^ is shown in Fig. 1.

These models were developed using six algorithms: *k*-nearest neighbor (kNN), decision tree, random forest, neural network, logistic regression, and gradient boosting.

Brief descriptions of the six ML models are as follows:

- The *k*NN algorithm^15^ is an ML instance-based model that stores all instances of the training dataset and makes predictions based on neighborhood proximity, as defined by a similarity metric.
- The decision tree algorithm^16^ is a tree-structured prediction model that starts with a root node and progresses to a leaf node. Each internal node represented a predictor variable, each internal node connection represented a choice, and each leaf node represented the outcome variable.
- The random forest algorithm^17^ is an ML ensemble model that combines multiple decision trees to achieve increased prediction accuracy. Each uncorrelated decision tree in the random forest makes a prediction, and the prediction with the largest number of votes is used as the final prediction for the algorithm.
- The neural network algorithm^18^ is an ML model that mimics the signal transmission through neurons in the human brain. The algorithm comprises multiple layers of nodes: an input layer, multiple hidden layers, and an output layer. Each node functions as a neuron, with a threshold value. If the collected signal reaches this threshold, the nodes are activated and the signal is transferred to the next layer in the network. Predictions were continuously generated until the signal reached the output layer.
- The logistic regression algorithm^19^ was used for binary and multiclass classifications. It utilizes a cost function, often known as a sigmoid function, to provide an estimate of probability values ranging from zero to one.
- The gradient boosting algorithm^20^ is another ensemble model that incorporates a large number of ML models to provide strong predictors. The algorithm uses a gradient boosting technique to calculate the residual error by training a simple base learner on all the training datasets. A new learner is then created to forecast the prior residual error and increase the accuracy of the prediction model.

### Internal evaluation

A tenfold cross-validation approach was employed for internal model validation. The dataset was randomly divided into 10 groups, with nine groups used for training and one for testing in each iteration. The average performance of the test results was subsequently used to assess the overall performance of the model across all the groups.

### Model comparison

The performance of all prediction models was assessed by comparing the area under the curve (AUC) using the Orange software. Additionally, calibration plots and mean Brier scores, calculated with the assistance of Python, were employed to evaluate the predictive ability and goodness of fit of the models. This facilitated the observation of agreement between the actual and predicted probabilities.

### External validation

The predictive models that exhibited outstanding performance, developed using the Cohort 1 dataset, were subsequently applied to the Cohort 2 dataset for external validation. Furthermore, the AUCs were computed to assess their performance in this independent dataset.

### Equation development

The intercepts and coefficients for the selected attributes across different outcomes were calculated using Orange software. Subsequently, we formulated the corresponding equations and developed estimators to predict the probabilities of various target outcomes.

## Results

### Study population and patient characteristics

A total of 8531 newborns were enrolled during the study period. However, 711 newborns were excluded due to missing data and 349 were excluded because they died within 12 h of delivery. Consequently, 7471 newborns with complete records were included in the final study. Cohort 1 and 2 included 6558 and 913 infants, respectively.

In Cohort 1 (Table 1), there was a significant difference (*p* < 0.05) between each variable and target outcome, except for: the use of prenatal MgSO4 between the group with and without severe IVH (*p* = 0.157); multiple births, across all outcomes (*p* = 0.671 in early mortality, *p* = 0.32 severe IVH, and *p* = 0.22 early poor outcomes); and congenital anomalies between the group with and without severe IVH (*p* = 0.76).

**Table 1:** Demographic data of the participants.

Variables	Cohort 1							Cohort 2						
		Early mortality		Severe IVH		Early poor outcomes			Early mortality		Severe IVH		Early poor outcomes	
	Total (N = 6558)	With (N = 282)	Without (N = 6276)	With (N = 533)	Without (N = 6025)	With (N = 719)	Without (N = 5839)	Total (N = 913)	With (N = 14)	Without (N = 899)	With (N = 58)	Without (N = 855)	With (N = 66)	Without (N = 847)
Antenatal steroid use, n (%)	5575 (85.0%)	225(79.8%)	5350 (85.3%)*	419 (78.6%)	5156 (85.6%)***	570 (79.3%)	5005 (85.7%)***	804 (88.1%)	14 (100%)	790 (87.9%)	47 (81.0%)	757 (88.5%)***	55 (83.3%)	749 (88.4%)***
Prenatal MgSO4 use, n (%)	3832 (58.4%)	147 (52.1%)	3685 (58.7%)*	296 (55.5%)	3536 (58.7%)	391 (54.4%)	3441 (58.9%)*	635 (69.6%)	12 (85.7%)	623 (69.3%)	36 (62.1%)	599 (70.1%)*	44 (66.7%)	591 (69.8%)*
PIH, n (%)	1831 (27.9%)	53 (18.8%)	1778 (28.3%)***	104 (19.5%)	1727 (28.7%)***	144 (20.0%)	1687 (28.9%)***	228 (25.0%)	3 (21.4%)	225 (25.0%)	5 (8.6%)	223 (26.1%)***	7 (10.6%)	221 (26.1%)**
Chorioamnionitis, n (%)	1004 (15.3%)	60 (21.3%)	944 (15.0%)**	138 (25.9%)	866 (14.4%)***	176 (24.5%)	828 (14.2%)***	151 (16.5%)	6 (42.9%)	145 (16.1%)**	23 (39.7%)	128 (15.0%)***	26 (39.4%)	125 (14.8%)***
GA, mean ± SD (weeks)	28.7 ± 3.0	25.3 ± 2.6	28.8 ± 3.0***	25.8 ± 2.4	28.9 ± 3.0***	25.8 ± 2.5	29.0 ± 2.9***	28.7 ± 3.0	24.5 ± 1.7	28.8 ± 2.9***	25.7 ± 2.2	28.9 ± 2.9***	25.5 ± 2.2	29.0 ± 2.9***
BBW < 500 g, n (%)	145 (2.2%)	48 (17.0%)	97 (1.6%)***	45 (8.4%)	100 (1.7%)**	75 (10.4%)	70 (1.2%)***	17 (1.9%)	1 (7.1%)	16 (1.8%)***	5 (8.6%)	12 (1.4%)***	5 (7.6%)	12 (1.4%)***
BBW 500–1000 g, n (%)	2482 (37.8%)	181 (64.2%)	2301 (36.7%)***	351 (65.9%)	2131 (35.4%)***	463 (64.4%)	2019 (34.6%)***	320 (35.0%)	12 (85.8%)	308 (34.3%)***	36 (62.1%)	284 (33.2%)***	44 (66.7%)	276 (32.6%)***
BBW 1000–1500 g, n (%)	3931 (60.0%)	53 (18.8%)	3878 (61.7%)***	137 (25.7%)	3794 (62.9%)***	181 (25.2%)	3750 (64.2%)***	576 (63.1%)	1 (7.1%)	575 (63.9%)***	17 (29.3%)	559 (65.4%)***	17 (25.7%)	559 (66.0%)***
Multiple births, n (%)	2052 (31.3%)	85 (30.1%)	1967 (31.3%)	156 (29.3%)	1896 (31.5%)	210 (29.2%)	1842 (31.6%)	292 (32.0%)	6 (42.9%)	286 (31.8%)	13 (22.4%)	279 (32.6%)	17 (25.8%)	275 (32.5%)
Cesarean section, n (%)	4879 (74.4%)	189 (67.0%)	4690 (74.7%)**	346 (64.9%)	4533 (75.2%)***	474 (65.9%)	4405 (75.4%)***	662 (72.5%)	9 (64.3%)	653 (72.6%)	32 (55.2%)	630 (73.7%)**	38 (57.6%)	624 (73.7%)**
Small for GA, n (%)	2729 (41.6%)	98 (34.8%)	2631 (41.9%)*	124 (23.3%)	2605 (43.2%)***	193 (26.8%)	2536 (43.4%)***	366 (40.1%)	4 (28.6%)	362 (40.3%)	13 (22.4%)	353 (41.3%)**	15 (22.7%)	351 (41.4%)**
Male gender, n (%)	3400 (51.9%)	168 (59.6%)	3232 (51.5%)**	314 (58.9%)	3086 (51.2%)***	423 (58.8%)	2977 (51.0%)***	461 (50.5%)	7 (50.0%)	454 (50.5%)	33 (56.9%)	428 (50.1%)***	37 (56.1%)	424 (50.1%)***
Apgar score < 7 at 1 min, n (%)	3814 (58.2%)	254 (90.1%)	3560 (56.7%)***	464 (87.1%)	3350 (55.6%)***	628 (87.3%)	3186 (54.6%)***	516 (56.5%)	14 (100%)	502 (55.8%)**	55 (94.8%)	461 (53.9%)***	63 (95.5%)	453 (53.5%)***
Apgar score < 7 at 5 min, n (%)	1118 (17.0%)	160 (56.7%)	958 (15.3%)***	233 (43.7%)	882 (14.6%)***	332 (46.2%)	786 (13.5%)***	148 (16.2%)	3 (21.4%)	145 (16.1%)**	22 (37.9%)	126 (14.7%)***	25 (37.9%)	123 (14.5%)***
BT (°C), mean ± SD	36.0 ± 0.9	35.4 ± 1.3	36.0 ± 0.9***	35.8 ± 1.2	36.0 ± 0.9***	35.7 ± 1.2	36.0 ± 0.9***	36.2 ± 0.7	35.4 ± 1.1	36.2 ± 0.7*	36.1 ± 1.0	36.2 ± 0.7*	36.0 ± 1.1	36.2 ± 0.7*
Early-onset sepsis, n (%)	169 (2.6%)	27 (9.6%)	142 (2.3%)***	41 (7.7%)	128 (2.1%)***	56 (7.8%)	113 (2.0%)***	24 (2.6%)	1 (7.1%)	23 (2.6%)	6 (10.3%)	18 (2.1%)**	6 (9.1%)	18 (2.1%)**
RDS, n (%)	4861 (74.1%)	274 (97.2%)	4587 (73.1%)***	490 (91.9%)	4371 (72.6%)***	670 (93.2%)	4191 (71.8%)***	729 (80.0%)	14 (100%)	715 (79.5%)**	56 (96.6%)	673 (78.7%)***	64 (97.0%)	665 (78.5%)***
Congenital anomalies, n (%)	124 (1.9%)	22 (7.8%)	102 (1.6%)***	11 (2.1%)	113 (1.9%)	30 (4.2%)	94 (1.6%)***	20 (2.2%)	0 (0%)	20 (2.2%)	3 (5.2%)	17 (2.0%)***	3 (4.6%)	17 (2.0%)***
Neonatal resuscitation, n (%)	6373 (97.2%)	282 (100%)	6091 (97.1%)**	532 (99.8%)	5841 (97.0%)***	718 (99.9%)	5655 (96.9%)***	899 (98.5%)	14 (100%)	885 (98.4%)	58 (100%)	841 (98.4%)***	66 (100%)	833 (98.4%)***
Oxygen supplementation, n (%)	6189 (94.4%)	278 (98.6%)	5911 (94.2%)**	520 (97.6%)	5669 (94.0%)***	704 (97.9%)	5485 (94.0%)***	877 (96.1%)	14 (100%)	863 (96.0%)	57 (98.3%)	820 (95.9%)***	65 (98.5%)	812 (95.9%)***
DRCPAP ventilation, n (%)	2225 (34.0%)	39 (13.8%)	2186 (34.8%)***	86 (16.1%)	2139 (35.5%)***	113 (15.7%)	2112 (36.2%)***	301 (33.0%)	1 (7.1%)	300 (33.4%)*	12 (20.7%)	289 (33.8%)*	12 (18.2%)	289 (34.1%)**
Positive pressure ventilation, n (%)	3455 (52.7%)	218 (77.3%)	3237 (51.6%)***	384 (72.1%)	3071 (51.0%)***	530 (73.7%)	2925 (50.1%)***	575 (63.0%)	14 (100%)	561 (62.4%)**	49 (84.5%)	526 (61.5%)***	57 (86.4%)	518 (61.2%)***
Endotracheal tube ventilation, n (%)	1976 (30.1%)	232 (82.3%)	1744 (27.8%)***	364 (68.3%)	1612 (26.8%)***	513 (71.4%)	1463 (25.1%)***	251 (27.5%)	11 (78.6%)	240 (26.7%)***	42 (72.4%)	209 (24.4%)***	50 (75.8%)	201 (23.7%)***
Chest compression, n (%)	303 (4.6%)	59 (20.9%)	244 (3.9%)***	57 (10.7%)	246 (4.1%)***	101 (14.1%)	202 (3.5%)***	18 (2.0%)	1 (7.1%)	17 (1.9%)	6 (10.3%)	12 (1.4%)***	7 (10.6%)	11 (1.3%)***
Epinephrine administration, n (%)	244 (3.7%)	51 (18.1%)	193 (3.1%)***	67 (12.6%)	177 (2.9%)***	99 (13.8%)	145 (2.5%)***	17 (1.9%)	0 (0%)	17 (1.9%)	4 (6.9%)	12 (1.4%)**	4 (6.1%)	10 (1.1%)*

*GA* gestational age, *BBW* birth body weight, *PIH* pregnancy-induced hypertension, *BT* body temperature, *RDS* respiratory distress syndrome, *DRCPAP* delivery room continuous positive airway pressure.

*P ≤ 0.05, **P ≤ 0.01, ***P ≤ 0.001.

In Cohort 2 (Table 1), there were no significant differences in antenatal steroid use, prenatal MgSO4 use, pregnancy-induced hypertension, multiple births, Cesarean section, small for GA, sex, early onset sepsis, congenital anomalies, neonatal resuscitation, oxygen supplement, chest compression, or epinephrine administration between infants with and without early mortality (*p* = 0.17, 0.19, 0.76, 0.38, 0.49, 0.38, 0.97, 0.29, 0.57, 0.64, 0.45, 0.16, 0.60, respectively). Similarly, there were no significant differences in multiple births between the group with and without severe IVH (*p* = 0.29) and with and without early poor outcomes (*p* = 0.20). The discrepancy observed, wherein significant differences were found between each variable and the target outcome in Cohort 1, whereas such differences were not apparent in Cohort 2, could potentially be attributed to the limited sample size of Cohort 2.

### Selection of predictors

**Figure 2:**
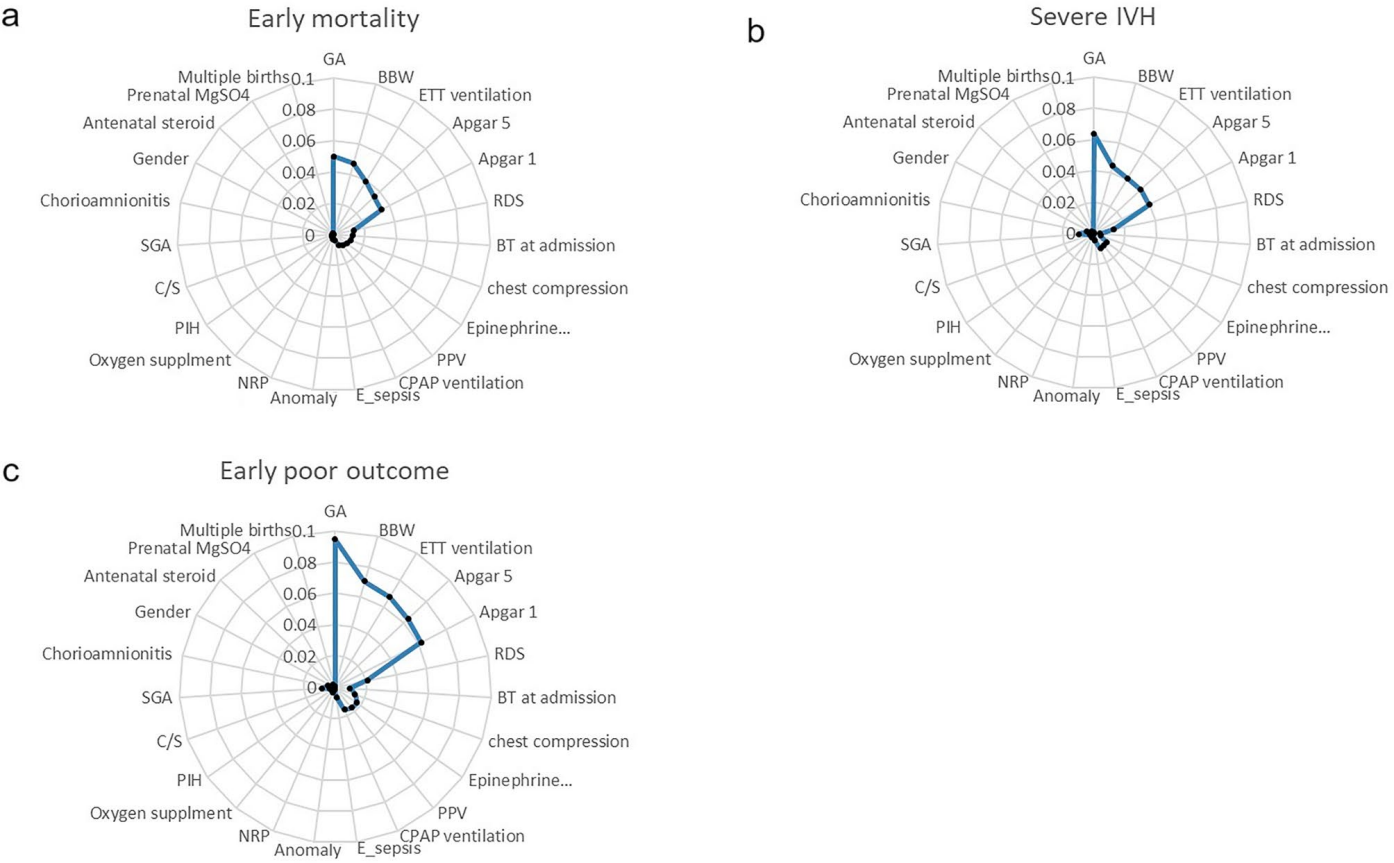
Radar charts of attribute selection with the information gain attribute evaluator. The top five critical variables on the radar chart are GA, BBW, endotracheal tube ventilation, 5-min Apgar score, and 1-min Apgar score. *GA* gestational age, *BBW* birth body weight, *ETT* endotracheal tube, *Apgar 5* 5-min Apgar score, *Apgar 1* 1-min Apgar score, *RDS* respiratory distress syndrome, *BT* body temperature, *epinephrine* epinephrine administration, *PPV* positive pressure ventilation, *CPAP* continuous positive airway pressure, *E_sepsis* early onset sepsis, *NRP* neonatal resuscitation, *PIH* pregnancy-induced hypertension, *C/S* Cesarean section, *SGA* small for gestational age.

Attribute selection, based on the Weka information gain attribute evaluator, enabled the condensed and generic application of the prediction models. The actual values generated by the evaluator for each variable were listed in Fig. 2 and Supplementary Table S1, revealing notable distinctions between the top five ranked variables and those ranked sixth and beyond. Furthermore, variables ranked second to fifth exhibited similar scores. Consequently, the initial selection included the top five variables: gestational age (GA), birth body weight (BBW), 1-min Apgar score, 5-min Apgar score, and endotracheal tube ventilation during initial resuscitation, for model development.

Additionally, considering collinearity concerns, further analysis was conducted using Variance Inflation Factor (VIF) values^21^ as presented in the Supplementary Table S2. This analysis indicated significant collinearity between the 1-min Apgar score and the 5-min Apgar score. Based on prior research^22^ The 5-min Apgar score is regarded as a more reliable predictor of neonatal outcomes compared to the 1-min Apgar score. Therefore, we opted to exclude the 1-min Apgar score from our prediction variables during model development.

### Model development and comparison

**Figure 3:**
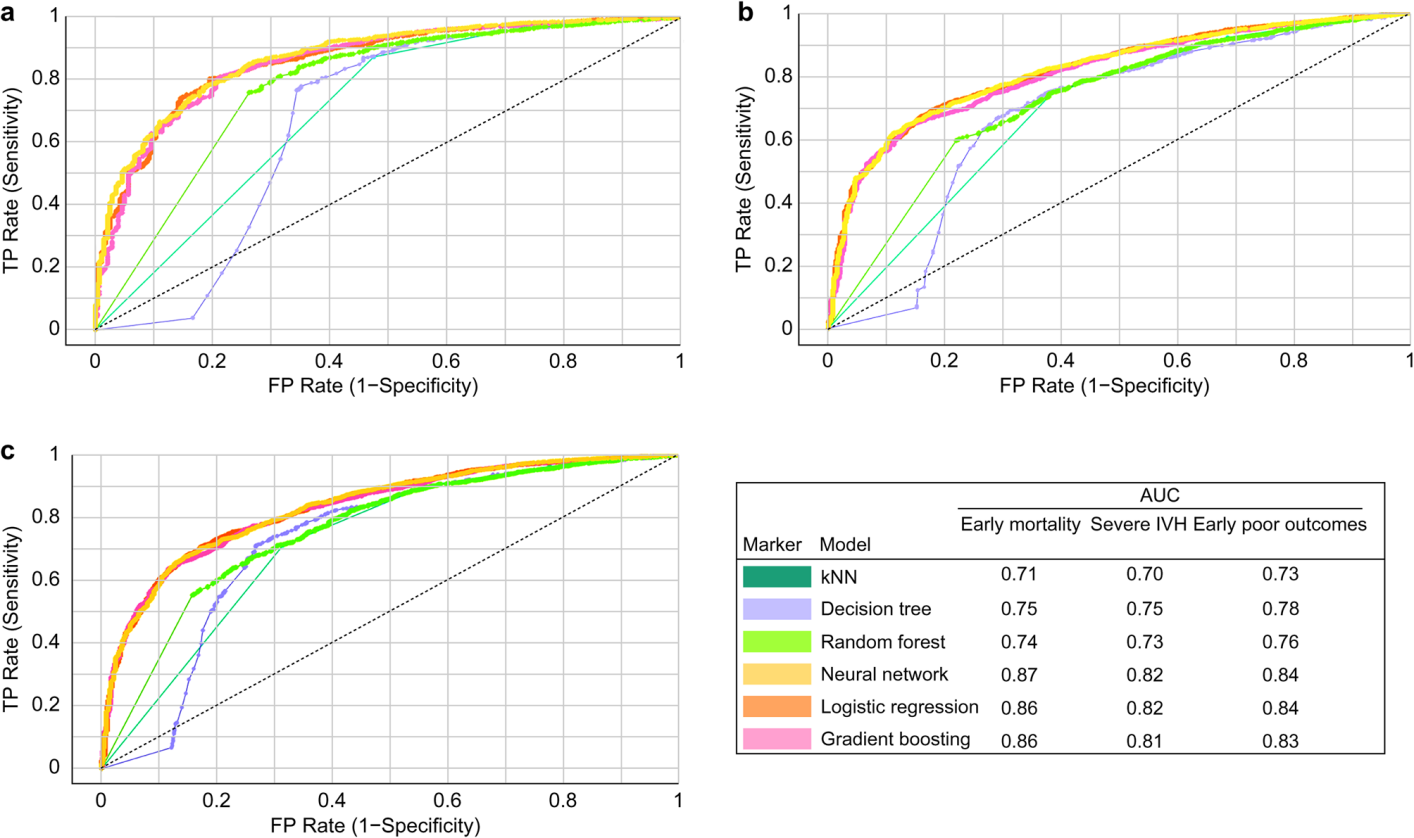
ROC curve analysis of six prediction models in the internal validation set. (**a**) ROC of early mortality; (**b**) ROC of severe IVH; (**c**) ROC of early poor outcomes. *ROC* receiver operating characteristic.

**Figure 4:**
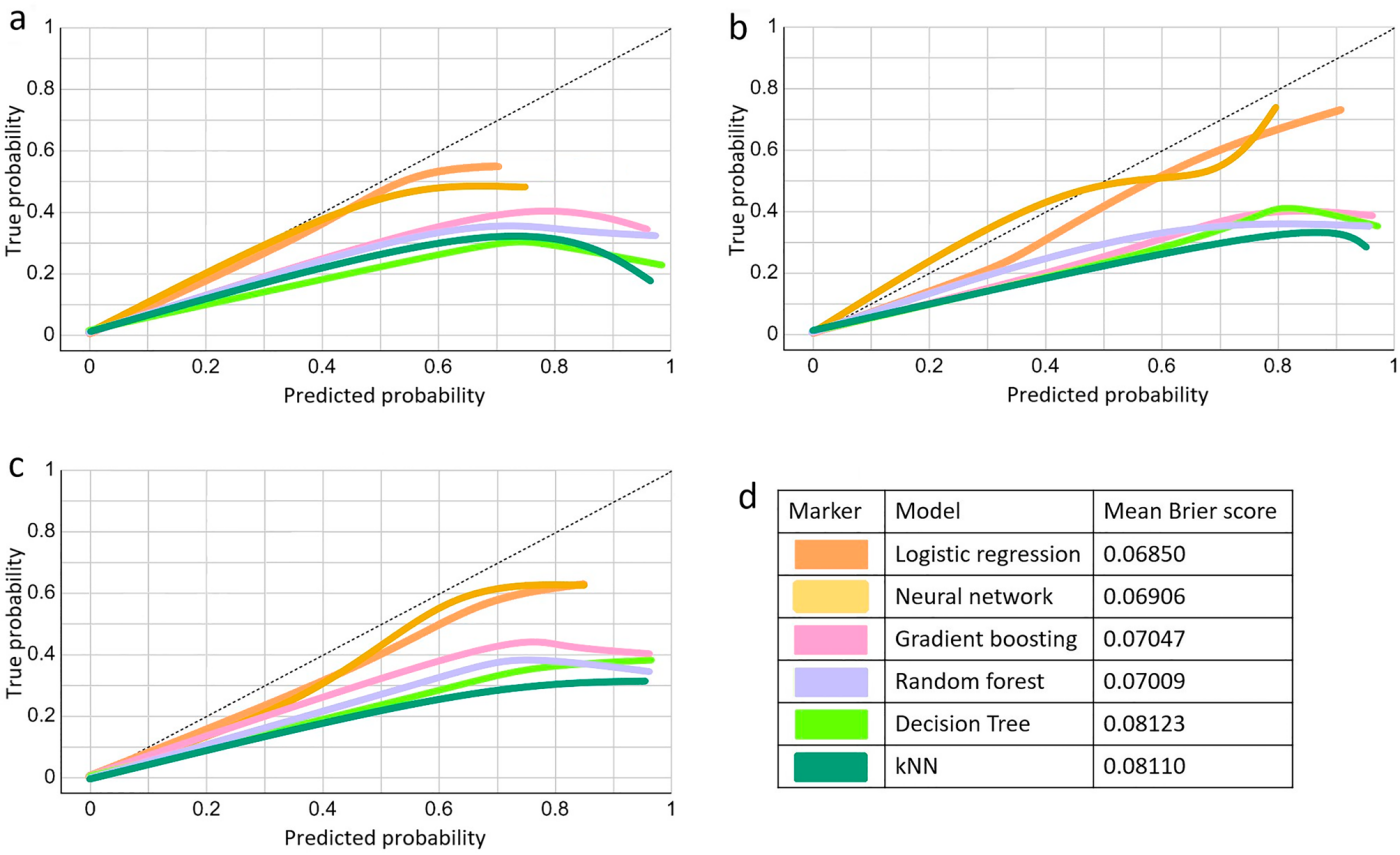
Calibration plot and mean Brier score of six prediction models in the internal validation set. (**a**) Calibration plot of early mortality. (**b**) Calibration plot of severe IVH. (**c**) Calibration plot of early poor outcomes. (**d**) Mean Brier score of three target outcomes.

The four most crucial variables, which were top-ranked and showed no significant collinearity, were utilized in the development of prediction models using Orange software. The internally validated receiver operating characteristic (ROC) curve results (Fig. 3) indicated that the neural network, logistic regression, and gradient boosting models were the most optimal predictive models for all target outcomes, with AUC values of 0.87, 0.86, and 0.86, respectively, for the prediction of early mortality; 0.82, 0.82, and 0.81, respectively, for severe IVH; and 0.84, 0.84, and 0.83, respectively, for early poor outcomes. The calibration plot illustrates the consistency between predictions and observations across different percentiles of predicted values. Comparing the calibration of all models through a scatter plot reveals the agreement between predictions and observations. According to Fig. 4, both logistic regression and neural network models demonstrated superior calibration performance, as depicted in the calibration plot. Furthermore, the logistic regression model achieved the best mean Brier score across three predictive outcomes, with a score of 0.0685, followed closely by the neural network model, which attained the lowest mean Brier Score of 0.06906. In contrast, the kNN and decision tree models exhibited less favorable calibration performance, with the highest mean Brier scores recorded at 0.0811 and 0.08123, respectively.

**Figure 5:**
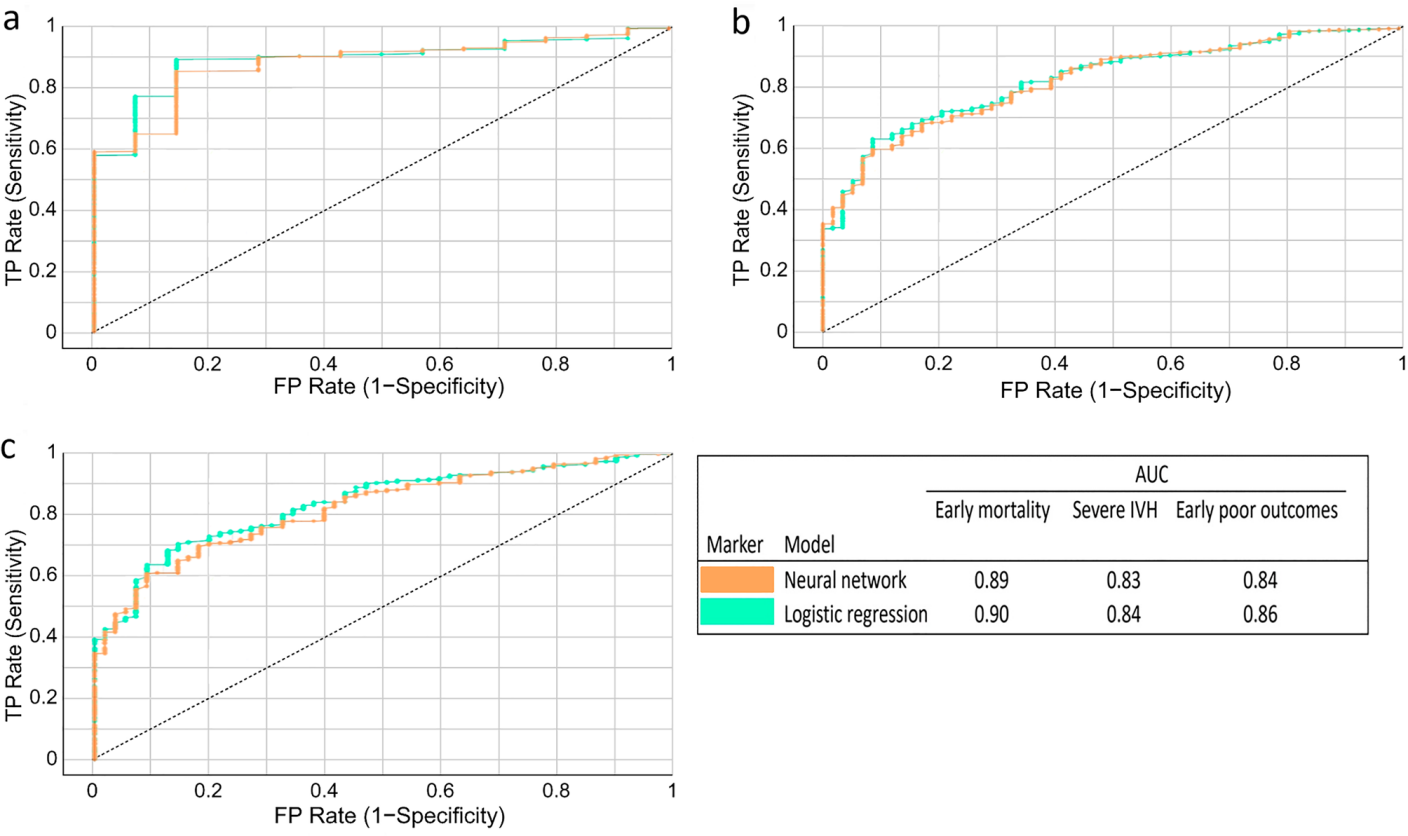
ROC curve analysis of three prediction models in the external validation set. (**a**) ROC of early mortality; (**b**) ROC of severe IVH; (**c**) ROC of early poor outcomes. *ROC* receiver operating characteristic.

For external validation by Cohort 2, we utilized the most powerful prediction models, namely logistic regression and neural network models. The results of the ROC curve analysis (Fig. 5) indicated exceptional predictive capabilities across all outcomes. Specifically, the AUC values were 0.90 and 0.89, respectively, for early mortality prediction; 0.84 and 0.83, respectively, for severe IVH prediction; and 0.86 and 0.84 for early poor outcome prediction for the logistic regression and neural network models, respectively.

### Equation development

We used Orange software to calculate the intercepts and coefficients necessary for constructing the prediction models through logistic regression. The results are summarized in Table 2. An equation was formulated for each target outcome as follows: outcome estimators suitable for clinical applications were developed using Microsoft Excel 2016.

**Table 2:** Intercept and coefficient values of the attributes in various models developed using logistic regression.

	Early mortality	Severe IVH	Early poor outcomes
Intercept	4.64898	7.08865	7.31688
GA	− 0.190294	− 0.303319	− 0.276669
BBW	− 0.180616	− 0.012063	− 0.0560051
5th-min Apgar score	− 0.156589	− 0.065139	− 0.126714
ETT ventilation	0.285209	0.18874	0.26231

*GA* gestational age, *BBW* birth body weight, *ETT* endotracheal tube.

As an illustrative example, consider a premature male infant born with a GA of 24 weeks and birth weight within the range of 601–700 g. The 5-min Apgar scores were 6, respectively. Importantly, intubation was not required during initial neonatal resuscitation in the delivery room. By inputting these parameters into the outcome estimator, we ascertained the following probabilities: 20% likelihood of early mortality, 35% likelihood of severe IVH, and 44% likelihood of early poor outcomes (Table 3).

**Table 3:** A table of the early poor outcomes estimator.

Outcomes evaluator			
Attributes		Outcomes	Probability (%)
Gestational age (weeks)	24	Early mortality	20
Birth body weight (g)	601–700	Severe intraventricular hemorrhage	35
Apgar 5	6	Early poor outcome	44
ETV	No		

## Discussion

In this study, we used a nationwide retrospective database comprising data on VLBW preterm infants and their associated variables collected immediately after their initial management in the delivery room. Our objective was to develop a predictive model for early mortality, severe IVH, and early poor outcomes using an -ML approach. Following the application of this approach, we identified GA, BBW, 5-min Apgar score, and intubation in the delivery room as the top four most crucial factors for constructing prediction models. Notably, we found that both the logistic regression and neural network models demonstrate superior performance, as indicated by their higher AUROC values. This suggests that they have better discriminative ability in distinguishing between different outcomes. Additionally, these models are well-calibrated, meaning that the predicted probabilities align closely with the observed frequencies of outcomes. Moreover, they have been effectively validated across different cohorts within this study, highlighting their robustness and generalizability across diverse populations or settings. Overall, the logistic regression and neural network models excel in terms of their high AUROC values, good calibration, and successful validation across various cohorts, making them reliable predictors of outcomes in this study.

Currently available scoring systems for predicting early mortality in neonates include: the Clinical Risk Index for Babies (CRIB) II^23^ Score for Neonatal Acute Physiology Perinatal Extension II (SNAPPE-II)^24^ and the Eunice Kennedy Shriver National Institute of Child Health and Human Development (NICHD) calculator^25^ for neonatal conditions or outcomes. These prediction models have been widely employed and subjected to external validation in multiple studies^26^

In our research, similar to CRIB II and NICHD, we identified GA and BBW as significant risk factors. A systematic review underscored the significance of these risk factors in neonatal mortality in neonatal intensive care units, with GA and BBW emerging as the most frequently cited contributors to neonatal mortality^27^ Additionally, an investigation conducted on the Taiwanese population, using data from birth certificates and death registries, established a robust correlation between GA, BBW, and the incidence of early mortality^28^

In 1952, Dr. Virginia Apgar pioneered the development of a scoring system designed to evaluate the physical condition of newborns and gauge their need for resuscitative interventions. Her groundbreaking work revealed a significant correlation between neonatal survival up to 28 days of age and the infant’s condition at delivery^29^ Notably, contemporary research has substantiated the enduring relevance of the Apgar score, reaffirming its significance nearly five decades later^30^

Although the Apgar score was initially conceived to assess term infants during an era characterized by high neonatal mortality rates among preterm infants, a recent investigation showed that the relative risk of neonatal mortality consistently escalates as the Apgar score diminishes across all GA categories^31^ Similarly, we included the Apgar score as a pivotal variable for outcome prediction in our study.

In our study, intubation emerged as the most important variable among all initial management procedures conducted in the delivery room. Notably, corroborative research conducted in countries such as Korea^32^ Iran^33^ Thailand^34^ and Brazil^35^ has similarly identified intubation as a pivotal risk factor for neonatal outcomes.

In our study, antenatal steroid administration and multiple births did not demonstrate statistical significance as variables for outcome prediction despite their inclusion in the NICHD calculator. This discrepancy may be attributed to the high prevalence of antenatal steroid administration in Taiwan, where 85% of the patients in our study received this treatment, in contrast to the population encompassed by the NICHD calculation, where approximately 70% received antenatal steroids. These demographic differences within the study population may have attenuated the influence of these variables on study outcomes.

In contrast, Boghossian et al.^36^ reported that the beneficial effects of antenatal steroids on mortality were statistically significant, primarily in infants born between 24 and 25 weeks of gestation. This observation suggests that the efficacy of antenatal steroids in reducing mortality may be contingent on GA.

Multiple births were associated with a notably elevated risk of mortality, particularly among extremely premature infants born at 26 weeks of gestation or earlier, as indicated in prior research^37^ In our study cohort, where the mean GA of the infants was 28.7 weeks, this characteristic may explain why antenatal steroid administration and multiple births were not significant factors in our analysis.

ML is a subset of artificial intelligence that has been extensively used in healthcare^38^ According to a recent systematic review^39^ concerning the deployment of ML models for forecasting neonatal mortality, prominent ML algorithms include neural networks, logistic regression, and random forests. The reviewed articles collectively reported a mean AUC range spanning from 58.3 to 97.0%, with the average exceeding 70%. These findings underscore the ability of ML models to predict neonatal mortality. In our ML -based predictive models, the AUC values demonstrated a comparable and laudable level of performance when juxtaposed with other ML-based models.

In the context of predicting IVH, it is noteworthy that all four variables incorporated into our predictor previously demonstrated strong predictive capabilities for IVH, with particular emphasis on GA. Furthermore, the significance of endotracheal tube ventilation has been underscored in the literature. Additionally, when comparing our IVH predictor to previous models (AUC 0.67–0.85 for severe IVH prediction), our predictor exhibits an outstanding performance^40^

Notably, despite external validation of the CRIB II, SNAPPE-II, and NICHD prediction models in diverse study populations, none of these models incorporated data from the Taiwanese population into their assessments. Predictive methodologies rely heavily on epidemiological population data to predict specific outcomes^41^ It is important to emphasize that the utility of a predictive model may be compromised by the possibility that the model is built upon data that could become outdated by the time it undergoes validation.

To the best of our knowledge, our predictive model represents a pioneering endeavor in the development of outcome-predictive models. This was the first initiative to construct such models based on the most current and comprehensive datasets available in Taiwan. Moreover, our model can predict early mortality, severe IVH, and early poor outcomes in VLBW preterm infants immediately following their initial management in the delivery room. Remarkably, this predictive capability was achieved using only four factors, eliminating the need for time-consuming blood sampling; however, these inherent advantages may facilitate widespread application in the Taiwanese population.

### Limitations

This study had several limitations. First, restrictions imposed by the available databases impeded the collection of precise clinical data such as blood pressure, oxygen demand, and comprehensive laboratory data encompassing hemograms, biochemical markers, and blood gas analyses. The inclusion of these clinical parameters could potentially enhance the predictive performance of the model^26,39^. Second, for privacy protection, the Taiwan neonatal network database recorded anonymous information, with gestational age rounded down and birth body weight recorded in ranges. These unavoidable limitations may impact the collinearity between variables. Third, while our prediction models demonstrated a high degree of accuracy in forecasting outcomes, they lack adaptability over time. As clinical dynamics evolve, these models may experience a decline in predictive accuracy. Fourth, variations in management and procedures across institutions may introduce potential biases that could be unavoidable in our study. Fifth, it is important to acknowledge that ML models may inadvertently manifest bias and discriminatory tendencies. Therefore, additional external validations across diverse population groups are required. This validation should explore whether the model generated can be applied with equal efficacy to populations other than Taiwanese cohorts to ensure a broader range of applicability.

## Conclusions

In this study, we developed an outcome predictor designed to predict early mortality, severe IVH, and early poor outcomes in preterm VLBW infants. This predictive model relied on the assessment of four readily available factors immediately after birth: GA, BBW, 5-min Apgar score, and endotracheal tube ventilation during initial resuscitation. Our analysis has yielded a formula that demonstrates exceptional performance, as evidenced by the high AUC values in both the internal validation cohort and the independent external validation population. Furthermore, it is well-calibrated, as evaluated by calibration plots and mean Brier scores. This prediction formula may prove to be a valuable tool and provide essential prognostic information for parents, aiding them in making informed decisions regarding the care and future of VLBW preterm infants. Furthermore, it may offer healthcare providers valuable guidance and facilitates the formulation of effective decision-making strategies for the clinical management of vulnerable infants. However, further validation across diverse populations is required to ensure broader applicability. Moreover, the inclusion of clinical parameters may further improve model accuracy.

### Supplementary Information

Supplementary Tables.

## Data Availability

According to the Taiwan Neonatal Network (TNN) Database Availability and Application Policy, although being anonymized and de-identified, the data are confidential. The data from TNN must only be available to individuals who have access for the authorized research. The data from this study are available from the corresponding author upon reasonable request.
