# Supplementary material for: Predicting early mortality and severe intraventricular hemorrhage in very-low birth weight preterm infants: a nationwide, multicenter study using machine learning
Source: Sci Rep. 2024 May 12;14:10833. doi: 10.1038/s41598-024-61749-1 (PMC11088707; doi:10.1038/s41598-024-61749-1)
Supplement: Supplementary file 1 — Supplementary Tables. [file 41598_2024_61749_MOESM1_ESM.docx]

**Supplementary Table S1.** Actual Variable Values for outcomes from Information Gain Evaluation

| Variable | Values | | |
| --- | --- | --- | --- |
|  | Early mortality | Severe IVH | Early poor outcomes |
| GA | 0.0500305 | 0.06354009 | 0.094468 |
| BBW | 0.0471274 | 0.04470988 | 0.070277 |
| Endotracheal tube ventilation | 0.0393615 | 0.04104996 | 0.067301 |
| 5th-min Apgar score | 0.035483 | 0.04098039 | 0.063913 |
| 1st-min Apgar score | 0.0343477 | 0.03996912 | 0.062243 |
| Respiratory distress syndrome | 0.013113 | 0.01297971 | 0.021207 |
| Body temperature | 0.0120272 | 0.00383795 | 0.009697 |
| Chest compression | 0.011442 | 0.00452606 | 0.013672 |
| Epinephrine administration | 0.0102142 | 0.00985118 | 0.017337 |
| Positive pressure ventilation | 0.0088974 | 0.01031844 | 0.017039 |
| DRCPAP ventilation | 0.007384 | 0.01059597 | 0.01559 |
| Early-onset sepsis | 0.0036516 | 0.00436502 | 0.006392 |
| Congenital anomalies | 0.0033729 | 0.00000688 | 0.001824 |
| Neonatal resuscitation | 0.0018102 | 0.00262063 | 0.003833 |
| Oxygen supplementation | 0.0015342 | 0.00151519 | 0.00267 |
| PIH | 0.0014006 | 0.00274948 | 0.003097 |
| Cesarean section | 0.0010655 | 0.00303085 | 0.003353 |
| Small for GA | 0.0008138 | 0.00943464 | 0.008377 |
| Chorioamnionitis | 0.0007828 | 0.00470025 | 0.004985 |
| Gender | 0.0007567 | 0.00131127 | 0.001744 |
| Antenatal steroid use | 0.0006335 | 0.00200203 | 0.00223 |
| Prenatal MgSO4 use | 0.0005011 | 0.00036332 | 0.000729 |
| Multiple births | 0.0000339 | 0.0001761 | 0.000224 |

Abbreviations: GA, gestational age; BBW, birth body weight; PIH, pregnancy-induced hypertension; DRCPAP, delivery room continuous positive airway pressure.

**Supplementary Table S2.**Variance Inflation Factor of variables

| Model | Collinearity stastistic | |
| --- | --- | --- |
|  | Tolerance | VIF |
| (Constant) |  |  |
| GA | 0.411 | 2.432 |
| BBW | 0.433 | 2.311 |
| 1st-min Apgar score | 0.258 | 3.875^✽^ |
| 5th-min Apgar score | 0.260 | 3.847^✽^ |
| ETT ventilation | 0.586 | 1.708 |

✽ collinearity

The table displays the relationship between Variance Inflation Factor values and collinearity. VIF values higher than 3.3 are considered indicative of collinearity.

Abbreviations: GA, gestational age; BBW, birth body weight; ETT, endotracheal tube
